# Residual Effects of Acute and Subchronic Zolpidem Treatments on Attentional Processes in Aged Female Rats

**DOI:** 10.1111/fcp.70067

**Published:** 2025-12-11

**Authors:** Marianne Leger, Michel Boulouard, Christophe Liet, Ben Grayson, Michael Harte, Joanna C. Neill, Marie‐Laure Bocca, Véronique Lelong‐Boulouard

**Affiliations:** ^1^ Normandie Université UNICAEN, INSERM, COMETE, CYCERON, CHU de Caen Caen France; ^2^ Manchester Pharmacy School, b‐neuro University of Manchester Manchester UK; ^3^ Department of Pharmacology University Hospital of Caen Normandie Caen France

**Keywords:** 5C‐CPT, attention, processing speed, rats, residual effect, zolpidem

## Abstract

**Rational:**

Zolpidem, a hypnotic Z‐drug commonly prescribed to promote sleep, is predominantly used over the age of 50. In this elderly population, adverse behavioral disturbances, impaired driving performance, and an increased risk of falls have been frequently reported. These concerns have raised questions about the residual adverse effects of zolpidem in older adults, particularly on cognitive processes such as executive and attentional functions.

**Objectives:**

This study aimed to investigate the residual effects of zolpidem on attentional performance following either acute or subchronic administration in aged rats.

**Methods:**

A zolpidem dose of 3 mg/kg and a 3‐h postadministration time point were selected based on pharmacokinetic data from the literature and a dose–response analysis of its locomotor effects in the open‐field test. Attentional performance was then assessed in aged female rats treated with either saline or zolpidem (acutely or for 7 days), using the 5‐choice continuous performance task (5C‐CPT).

**Results:**

Acute zolpidem administration significantly reduced the percentage of correct responses, increased correct response latency, and showed a trend toward more omissions, indicative of impaired attentional performance and psychomotor slowing. These effects were not further observed after subchronic treatment, suggesting a potential tolerance over time.

**Conclusion:**

Our findings highlight a critical period of vulnerability following the initiation of zolpidem treatment, during which residual cognitive impairments may emerge. Such effects may compromise complex tasks requiring sustained attention and processing speed, such as driving, especially in older adults.

**Abbreviations**
ANOVA: analysis of variance
GABA: gamma‐aminobutyric acid
i.p.: intraperitoneally
SEM: standard error of the mean
5C‐CPT: 5‐choice continuous performance task
5C‐SRTT: 5‐choice serial reaction time task

## 1 Introduction

Since the coronavirus disease 2019 (COVID‐19) pandemic period, many people have developed psychiatric disorders such as depression or anxiety. This event further increased the number of people suffering from insomnia and the incidence of patients using hypnotic drugs, especially in the elderly population [1, 2]. These drugs are mainly prescribed to induce sedation or promote sleep in patients suffering from insomnia or complaining of poor sleep [3, 4]. Among hypnotic drugs, the Z‐drug zolpidem (marketed in the 1980s) is a benzodiazepine‐like drug (imidazopyridine) with sedative and hypnotic properties and exhibiting similar pharmacological characteristics to benzodiazepines. It is characterized by gamma‐aminobutyric acid (GABA) agonist activity and high selectivity for benzodiazepine ω1 (type 1) receptors, which correspond to GABA_A_ receptors containing α1 subunits [5, 6]. Due to its short half‐life, i.e., between 2 and 3 h in humans, and the few adverse effects reported by both epidemiology and experimental studies [7, 8], this drug was considered safe during two decades, despite the risks of dependence. However, since the early 2000s, emerging data on the risk of abuse and misuse [5, 9, 10, 11], along with innovative findings highlighting the next‐day deleterious effects on driving behavior and on walking [12, 13, 14, 15, 16], have raised concerns regarding the widespread use of zolpidem among hypnotic drugs. Nevertheless, despite changes in legislation regarding its prescription in some countries, such as France since 2017, zolpidem remains one of the most prescribed hypnotic drugs in the world [17, 18].

Concerning the deleterious residual effect of zolpidem, epidemiological studies have highlighted an increased risk of accidents both during the first week after prescription [19] and after 5 months [20]. An increased risk of inpatient falls after zolpidem intake has also been described [14]. Elderly patients appear to be especially susceptible to the deleterious effects of zolpidem. Indeed, residual effects were observed on driving performance in aged subjects but not in young ones [12, 21]. Moreover, a clinical study conducted on healthy volunteers reported both pharmacokinetic and pharmacodynamic differences related to gender following sublingual zolpidem administration, with higher plasma concentrations and greater effects observed in women compared to men [22], suggesting that women should be more sensitive than men. As mobility such as driving and walking requires sufficient cognitive, visual, and motor skills [23, 24, 25], investigating these skills after zolpidem use should be useful to understand the effects of this drug. Two recent meta‐analyses show conflicting results regarding the impact of zolpidem on cognitive functions [26, 27]. However, both warn about the acute cognitive effects of zolpidem, including memory and attentional impairments the next morning after bedtime intake.

In terms of clinical studies, numerous data have demonstrated dose‐ and delay‐dependent residual impairments after isolated intake of zolpidem in healthy volunteers or young patients [28, 29, 30, 31, 32]. However, to our knowledge, this has never been demonstrated in studies examining repeated doses in young or elderly individuals [33, 34, 35].

In rats, zolpidem also exerts hypnotic effects by acting on GABA_A_ receptors [36, 37]. GABA_A_ receptors are widely distributed in the rat brain, including the prefrontal cortex [38]. As in humans, numerous studies have focused on the cognitive effects of zolpidem after acute administration [39, 40, 41, 42, 43]. The data from all these studies, performed in young and middle‐aged rodents, suggest a possible alteration of attentional and executive processes after a single intake of zolpidem. Additionally, a few studies have examined the behavioral effects of zolpidem after chronic administration, particularly regarding the sedative residual effect, but none have focused on its impact on cognitive and attentional functions in young or aged animals [44, 45, 46].

In this context, the aim of the present study was to explore the residual behavioral effects of acute and subchronic zolpidem administration on attentional and executive processes in aging female rats. To this end, two behavioral experiments were performed in aged animals. The first experiment used the open field to determine the threshold dose of zolpidem that does not alter locomotor activity, thus ensuring the absence of sedative interference. The second experiment assessed the residual effects of zolpidem at the selected dose (3 mg/kg) on attentional performance using the 5‐Choice Continuous Performance Task (5C‐CPT), a validated paradigm for measuring vigilance, sustained and selective attention, as well as executive function in rats [47, 48, 49].

## 2 Materials and Methods

### 2.1 Subjects

A total of 60 female Lister‐Hooded rats (Charles River) were used (22‐month‐old; mean weight: 293 g). All animals were housed in groups of five under a standard light–dark cycle (lights on at 7:00 am) in a temperature (21°C ± 2°C) and humidity (55% ± 5%) controlled environment, with free access to food and water. Prior to the start of behavioral experiments, animals were food‐restricted to reduce their body weight to 90% of baseline. Rats had free access to water in their home cages. All experiments were conducted in accordance with the UK Animals (Scientific Procedures) Act 1986 and the European Communities Council Directive (2010/63/UE) regarding the care and use of animals for experimental procedures. Ethical approval was obtained from the University of Manchester ethical review panel (United Kingdom).

### 2.2 Drug Treatment

For all experiments, rats were randomly assigned to receive either a vehicle (0.9% saline) or zolpidem (Sigma‐Aldrich, France) solution administered intraperitoneally (i.p.) at a volume of 2 mL/kg. Zolpidem was dissolved in a solution of 5% Tween20/0.9% saline.

Experiment 1 was designed to determine, 3 h after acute i.p. administration, the doses of zolpidem not producing a sedative effect. The 3‐h time point was selected based on the known pharmacokinetic profile of zolpidem, a hypnotic compound characterized by rapid brain penetration and fast elimination, typically within 3 h following acute administration in rats [50]. The dose range tested was determined according to the literature data in rats [40, 51, 52].

### 2.3 Behavioral Studies

#### 2.3.1 Experiment 1: Selection of the Zolpidem Dose in the Open‐Field Test

Four groups of aged rats (*n* = 10 per group) received a single acute injection of either vehicle or zolpidem at doses of 1, 3, or 6 mg/kg. Residual effects of zolpidem on locomotor activity were assessed 3‐h postadministration using an open‐field test. Rats were individually placed in a black wooden box (0.7 m × 0.7 m × 0.4 m) with a grid of nine equal squares marked on the floor. The number of line crossings and rearings were measured during a 5‐min session to evaluate horizontal and vertical locomotor activity, respectively.

#### 2.3.2 Experiment 2: Residual Effects of Zolpidem (3 mg/kg) on Attention in the 5C‐CPT

Two groups of aged rats (*n* = 10 per group) were subchronically treated with either vehicle or zolpidem (3 mg/kg) via daily i.p. injections for seven consecutive days, administered at a fixed time (09:00 a.m.). The 3 mg/kg dose was selected based on the results of Experiment 1. Behavioral testing in the 5C‐CPT was conducted on the same animals following acute and subchronic treatment with either vehicle or zolpidem. All test sessions were initiated 3 h postinjection.

As already described in detail by Barnes et al. [48, 49], the apparatus consisted of eight sound‐attenuating dark chambers (25 × 25 cm; Campden Instruments Ltd., UK), each equipped with five apertures (2.5 cm^2^, 4 cm deep and positioned 2 cm above floor level) on the rear wall. A white LED was located at the rear of each aperture to deliver the light stimulus. Nose‐poke responses into the apertures were registered by infrared photocell beams. Food rewards (45‐mg Rodent Pellet, Test Diet, MO, USA) were delivered into a food magazine located on the front wall of the chamber. All chambers were connected to a PC, and data collection and analysis were managed by Abet II Touch software (Lafayette Instrument Company, IL, USA).

Training was conducted as previously described [48, 49, 53]. Briefly, rats were trained to detect the brief light stimulus presented pseudorandomly in one of the five apertures following variable inter‐trial intervals (4.0, 4.5, 5.5, and 6.0 s). A nose poke into the correct aperture within a limited hold period of 2 s was considered a correct response and was rewarded with a food pellet. The next inter‐trial interval was initiated immediately after food collection. An incorrect response (nose poke into the wrong aperture) or an omission (absence of response) resulted in a 5‐s time‐out, during which the chamber was illuminated and no reward was delivered. Rats were also exposed to the time‐out period after a nose poke made before the light stimulus onset (premature response) or repeated nose pokes into any apertures. During nontarget trials (in which lights were presented simultaneously in all apertures), rats were trained to withhold responding. Correct inhibition was rewarded, whereas a nose poke during these trials (false alarm) resulted in a time‐out. The same behavioral parameters as described previously by Barnes et al. [48, 49, 53] were measured or calculated. Training was considered successful when rats reached performance criteria of > 70% accuracy, < 25% omissions, and > 60% correct response. A session ended after 120 trials or when 30 mins had elapsed.

### 2.4 Statistical Analysis

Statistical analyses and graphical representations (mean ± standard error of the mean (SEM)) were performed using RStudio and GraphPadPrism. Behavioral data were first tested for normality (Shapiro–Wilk's test) and for homogeneity of variance (Levene's test). When these assumptions were not met, nonparametric tests were applied and results were expressed as boxplots (median ± quartiles). In Experiment 1, a one‐way analysis of variance (ANOVA) with drug treatment (vehicle or zolpidem) as the main factor was performed to investigate the influence of zolpidem on the number of line crossings and rearings in the open‐field test. When significant effects were observed, post hoc Tukey tests were conducted. In Experiment 2, a one‐way ANOVA with drug treatment (vehicle or zolpidem) as the main factor was performed for each parameter of the 5C‐CPT following either acute or subchronic treatment. For data that did not meet the assumptions of normality and homogeneity of variance, the nonparametric Mann–Whitney *U* test was applied. Statistical significance was set at *p* < 0.05.

## 3 Results

### 3.1 Experiment 1: Selection of the Zolpidem Dose Using the Open‐Field Test

**FIGURE 1:**
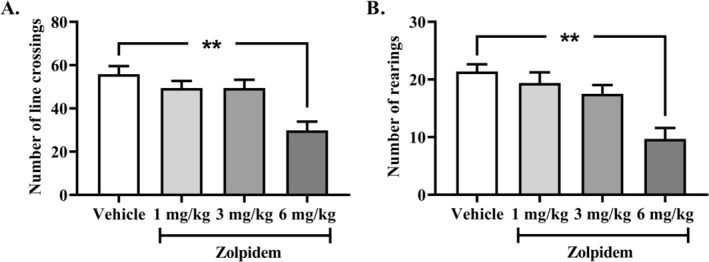
Effect of acute zolpidem administration (1, 3 or 6 mg/kg; i.p.) on locomotor activity assessed 3 h postinjection in the open‐field test. (A) Number of line crossings and (B) Number of rearings. Data are presented as mean ± SEM (*n* = 10 per group). One‐way ANOVA followed by Tukey post hoc analysis: **p < 0.01, significantly reduced compared to the vehicle group.

Residual effects of zolpidem (1, 3, 6 mg/kg; acute i.p. injection) on locomotor activity were assessed using the open‐field test, 3 h postinjection in aged rats. A significant effect of drug treatment was observed on both the number of line crossings and rearings (one‐way ANOVA: *F*_(3, 36)_ = 9.04, *p* < 0.001 and *F*_(3, 36)_ = 9.66, *p* < 0.001, respectively). Zolpidem at 6 mg/kg significantly reduced both horizontal and vertical locomotor activity compared to the vehicle group (Tukey post hoc analysis: *p* < 0.01; Figure 1A,B), suggesting a sedative effect. As 3 mg/kg was the highest dose that did not induce sedative effects, it was selected for use in Experiment 2.

### 3.2 Experiment 2: Residual Effects of Zolpidem (3 mg/kg) on Attention in the 5C‐CPT

**FIGURE 2:**
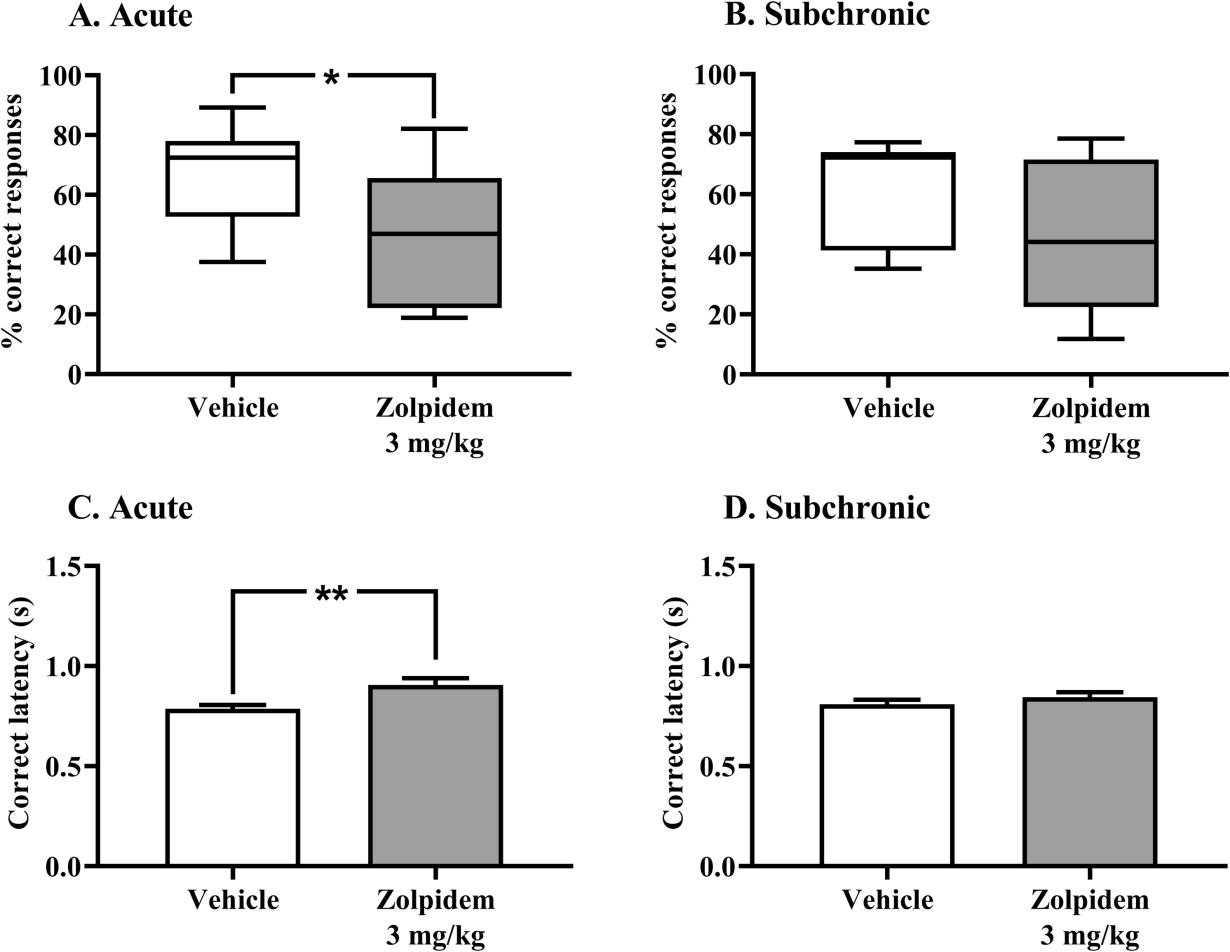
Residual effects of zolpidem administration (3 mg/kg; i.p.) on 5C‐CPT performance, assessed 3 h postinjection. (A and B) Percentage of correct responses following acute and subchronic zolpidem treatment. (C and D) Correct response latency following acute and subchronic zolpidem treatment. Data are presented as mean ± SEM (*n* = 10 per group). One‐way ANOVA: *p < 0.05; **p < 0.01, significantly different compared to the vehicle group.

Residual effects of zolpidem (3 mg/kg; acute or subchronic i.p. injection) on attentional performance were assessed using the 5C‐CPT, 3 h postinjection in aged rats. Compared to the vehicle group, the percentage of correct responses was significantly reduced following acute zolpidem administration (one‐way ANOVA: *F*_(1, 18)_ = 5.24, *p* < 0.05, Figure 2A, Table 1). A reduction in the percentage of correct responses was also observed following subchronic treatment with zolpidem, although this difference did not reach statistical significance (Mann–Whitney test: *U* = 29.00, *p* > 0.05, Figure 2B, Table 1).

**TABLE 1:** Residual effects of acute or subchronic zolpidem administration (3 mg/kg; i.p.) on 5C‐CPT performances, assessed 3 h postinjection. Data are expressed as mean ± SEM or median ± [Quartile 1/Quartile 3], depending on data distribution (*n* = 10 per group). One‐way ANOVA: *p < 0.05; **p < 0.01, significantly different compared to the vehicle group.

		Acute treatment		Subchronic treatment	
	Correlated behavior	Vehicle	Zolpidem	Vehicle	Zolpidem
**Measures (target trials)**					
Processed trials		109.0 [89.8;119.3]	120.0 [113.3;120.0]	118.5 [106.0;120.0]	114.5 [109.0;120.0]
Accuracy (%)	Selective attention	99.1 [92.9;100.0]	92.3 [72.9;100.0]	93.7 [89.4;97.1]	88.6 [74.8;95.2]
Correct responses (%)	General performance task	72.5 [52.7;78.1]	46.9 [22.1;65.6] *p < 0.05	72.2 [41.3;74.0]	44.1 [22.5;71.6]
Omissions (%)	Sustained attention	30.8 ± 5.6	48.0 ± 6.8 *p* = 0.054	34.7 ± 6.4	48.9 ± 8.3
Correct response latency (s)	Psychomotor speed	0.8 ± 0.0	0.9 ± 0.0 **p < 0.01	0.8 ± 0.0	0.8 ± 0.0
Incorrect latency (s)	General response speed	0.5 [0.0;1.0]	0.9 [0.0;1.2]	1.0 [0.6;1.2]	1.1 [0.8;1.2]
Magazine latency (s)	Motivational state	1.0 [0.9;1.4]	1.3 [1.1;1.9]	1.2 [1.0;1.6]	1.5 [1.1;1.7]
Premature responses	Motor impulsivity	6.0 [3.0;29.3]	7.5 [2.0;17.3]	5.0 [4.0;15.5]	7.5 [2.8;13.3]
**Measures (Nontarget trials)**					
Correct rejections (%)	Response inhibition	57.5 [41.2;74.4]	71.4 [54.9;82.4]	63.7 [47.5;83.2]	71.1 [63.2;82.2]
False alarm (%)	Incorrect response to nontarget trial	42.5 [25.6;58.8]	28.6 [17.6;45.1]	36.3 [16.8;52.6]	28.9 [17.8;36.8]
Sensitivity index	Vigilance	0.3 ± 0.1	0.2 ± 0.1	0.3 ± 0.1	0.1 ± 0.1
Responsivity index	Response strategy	0.1 ± 0.1	−0.2 ± 0.2	0.0 ± 0.2	−0.2 ± 0.1

Acute, but not subchronic, zolpidem administration also significantly increased correct response latency (one‐way ANOVA: *F*_(1, 18)_ = 9.04, *p* < 0.01 and *F*_(1, 18)_ = 1.20, *p* > 0.05, Figure 2C,D, Table 1) and produced a trend toward a higher percentage of omissions (one‐way ANOVA: *F*_(1, 18)_ = 4.26, *p* = 0.054, Table 1) compared to the vehicle group. No other parameters were significantly affected by either acute or subchronic zolpidem administration (Table 1).

## 4 Discussion

This study is the first to investigate the effects of zolpidem in aged rats trained in the 5C‐CPT, a paradigm designed to assess attentional processes and executive functions. Zolpidem significantly reduced the percentage of correct responses, increased correct response latency, and showed a trend toward more omissions, 3 h after acute administration in aged female rats. These findings suggest an acute impairment in attentional performance, which appears less pronounced following subchronic administration.

Before assessing the residual effects of zolpidem on attentional performance in the 5C‐CPT, we first evaluated the impact of increasing doses on locomotor activity using the open‐field test to determine the dose that could be used without inducing sedation. Among the three doses tested (1, 3, and 6 mg/kg), the 6 mg/kg dose was found to reduce locomotor and/or exploratory activity 3 h postadministration, suggesting a potential residual sedative effect. This result is in agreement with previous studies that found similar reductions in spontaneous locomotion [46, 54]. For instance, consistent decreases in locomotor activity were observed following a single administration of zolpidem at doses as low as 1 mg/kg in male Wistar rats [55, 56, 57]. However, these studies assessed locomotor activity within 20 to 30 min postinjection, corresponding approximately to peak plasma concentrations [58], which did not explore residual effects of zolpidem over longer time frames. In our study, the highest dose free from sedative effect was 3 mg/kg. This dose was therefore selected to assess the residual effects of zolpidem on attentional processes in the 5C‐CPT.

We investigated the effects of zolpidem in aged female rats using the 5C‐CPT, a task specifically designed to assess distinct attentional processes and executive functions [48, 59]. A key advantage of the 5C‐CPT is its cross‐species validity, as it has been adapted to both rodents and humans, thereby enabling translational research [47]. Initially developed in rodents to explore the brain substrates and neurotransmitter systems involved in attention and executive functions [59, 60], the 5C‐CPT has since been used in pharmacological research to develop rodent models of attentional deficits and to gain deeper insights into the neurochemical pathways underlying attentional processes [53, 61]. For example, 5C‐CPT performance was impaired in mice following administration of scopolamine, a nonselective muscarinic receptor antagonist [62], while amphetamine elicited proattentional effects [63]. By assessing, for the first time, the effects of both acute and subchronic zolpidem administration in aged rats using this task, our study contributes new insights into the pharmacological modulation of attentional processes. Indeed, zolpidem targets GABA_A_ receptors containing α1 subunits, which are widely expressed in key brain regions involved in attention and executive function, including the prefrontal cortex, thalamus, striatum, and hippocampus [64]. Through its action on these structures, zolpidem may impact various cognitive functions such as sustained attention, directed attention, or cognitive flexibility [65].

In the 5C‐CPT, a single acute administration of zolpidem (3 mg/kg) resulted in impairments in two performance parameters assessed 3 h postinjection. Specifically, compared to controls, the percentage of correct responses significantly decreases, and the correct response latency significantly increases, suggesting a residual impact of zolpidem on both general performance and psychomotor speed. However, as neither accuracy nor omission rate was significantly affected, it remains difficult to determine which specific attentional process was disrupted. We hypothesize a potential impairment of sustained attention, supported by a trend toward increased omissions combined with psychomotor slowing. Supporting this interpretation, three GABA‐related antiepileptic drugs (triazolam, phenobarbital, and chlordiazepoxide) were evaluated in another attentional task (the 5‐choice serial reaction time task, 5C‐SRTT). At the time of peak plasma concentration, these drugs produced dose‐related disruptions in attentional parameters in male Sprague–Dawley rats [66]. Moreover, deleterious residual effects of evening intake of zolpidem (10 and 20 mg) on speed of processing have been reported in human studies using the Digit Symbol Substitution Test [67] and the Stroop Color‐Word test [68]. In this test, an increase in omissions is sometimes interpreted as a decrease in motivation in treated animals [69]. Given the sedative properties of zolpidem, this hypothesis cannot be entirely ruled out as a potential explanation for the trend toward increased omissions observed in acute conditions. Nevertheless, it should be noted that, in our study, the decline in total correct responses and the trend toward more omissions occurred without changes in concerning magazine latency, sensitivity, or responsivity indices. In addition, the 3‐mg/kg dose of zolpidem used did not induce a sedative effect in the open field test. Taken together, and in line with previous pharmacological findings in the 5C‐SRTT [70], these data suggest that the observed impairment likely reflects a genuine attentional deficit rather than alterations in motivation, vigilance, or response strategies.

The residual attentional effects observed after a single administration of zolpidem were no longer detectable following a 7‐day subchronic treatment. Although a decrease in the percentage of correct responses and an increase in omissions persisted, these effects were no longer statistically significant. As previously described for other effects of zolpidem, this attenuation over time may be related to adaptive physiological mechanisms such as tolerance. Tolerance to the sedative and anticonvulsant properties of zolpidem has indeed been reported in mice treated with 5 mg/kg twice daily for 10 days [71]. Interestingly, Trenque et al. [58] reported no changes in pharmacokinetic parameters following acute or chronic administration (28 days) of zolpidem 5 mg/kg in rats. In contrast, an in situ hybridization study showed that a 14‐day treatment with zolpidem (15 mg/kg) significantly reduced the cortical expression of GABA_A_ α1 subunit mRNA in rats [36]. These findings suggest that the tolerance observed following subchronic zolpidem administration is likely driven by pharmacodynamic adaptations, such as a reduction in the availability of its molecular targets.

In this study, given the increased sensitivity of older individuals to the deleterious effects of zolpidem, we deliberately used aged rats. In this context, we assumed that age‐related pharmacokinetic changes would likely enhance the deleterious effects of zolpidem on attentional processes, particularly through drug accumulation [12, 50, 58]. However, it is also possible that certain age‐related pharmacodynamic modifications may have, conversely, limited the alterations observed under subchronic conditions. Indeed, clusters of reduced GABA‐A/BDZ receptor binding have been reported in various brain regions involved in cognitive processes, particularly in the frontal cortex, a region strongly implicated in attentional control [72, 73].

## 5 Conclusion

In conclusion, we highlighted for the first time residual attentional effects of zolpidem in aged rats trained in the 5C‐CPT following a single administration. As observed with other pharmacological effects of zolpidem, these impairments appear to diminish with repeated administration, likely due to adaptive brain mechanisms. The observed deficits in attentional processes and processing speed underscore the importance of the initiation period of zolpidem treatment as a critical window of vulnerability. These findings suggest that complex cognitive activities requiring high levels of attention and sustained processing speed, such as car driving, may be impaired, especially in older women.

## Author Contributions

**Leger Marianne:** conceptualization, investigation, formal analysis, visualization, writing – original draft, writing – review and editing. **Boulouard Michel:** conceptualization, writing – original draft, writing – review and editing. **Liet Christophe:** conceptualization, investigation, writing – original draft. **Grayson Ben:** methodology, investigation, resources, writing – review and editing. **Harte Michael:** methodology, project administration, resources, writing – review and editing. **Neill Joanna C.:** conceptualization, project administration, resources, writing – review and editing. **Bocca Marie‐Laure:** conceptualization, methodology, project administration, supervision, writing – original draft, writing – review and editing. **Lelong‐Boulouard Véronique:** conceptualization, methodology, project administration, supervision, writing – original draft, writing – review and editing.

## Funding

Christophe Liet was supported by a research grant from the French Ministry of Higher Education and Research.

## Conflicts of Interest

The authors declare no conflicts of interest.

## Data Availability

The data that support the findings of this study are available from the corresponding author upon reasonable request.
